# Use of Deep Learning to Evaluate Tumor Microenvironmental Features for Prediction of Colon Cancer Recurrence

**DOI:** 10.1158/2767-9764.CRC-24-0031

**Published:** 2024-05-23

**Authors:** Frank A. Sinicrope, Garth D. Nelson, Bahar Saberzadeh-Ardestani, Diana I. Segovia, Rondell P. Graham, Christina Wu, Catherine E. Hagen, Sameer Shivji, Paul Savage, Dan D. Buchanan, Mark A. Jenkins, Amanda I. Phipps, Carol Swallow, Loic LeMarchand, Steven Gallinger, Robert C. Grant, Reetesh K. Pai, Stephen N. Sinicrope, Dongyao Yan, Kandavel Shanmugam, James Conner, David P. Cyr, Richard Kirsch, Imon Banerjee, Steve R. Alberts, Qian Shi, Rish K. Pai

**Affiliations:** 1 Departments of Medicine and Oncology, Rochester, Minnesota.; 2 Gastrointestinal Research Unit, Mayo Clinic, Rochester, Minnesota.; 3 Division of Clinical Trials and Biostatistics, Mayo Clinic, Rochester, Minnesota.; 4 Department of Quantitative Health Sciences, Mayo Clinic, Rochester, Minnesota.; 5 Department of Laboratory Medicine and Pathology, Mayo Clinic, Rochester, Minnesota.; 6 Division of Medical Oncology, Mayo Clinic, Phoenix, Arizona.; 7 Department of Pathology, Mount Sinai Hospital, Toronto, Ontario, Canada.; 8 Mount Sinai Hospital, Toronto, Ontario, Canada.; 9 Colorectal Oncogenomics Group, Department of Clinical Pathology, The University of Melbourne, Parkville, Victoria, Australia.; 10 University of Melbourne Centre for Cancer Research, Victorian Comprehensive Cancer Centre, Parkville, Victoria, Australia.; 11 Genetic Medicine and Family Cancer Clinic, Royal Melbourne Hospital, Parkville, Victoria, Australia.; 12 Centre for Epidemiology and Biostatistics, Melbourne School of Population and Global Health, The University of Melbourne, Melbourne, Victoria, Australia.; 13 Public Health Sciences Division, Fred Hutchinson Cancer Research Center, Seattle, Washington.; 14 Department of Epidemiology, University of Washington, Seattle, Washington.; 15 Lunenfeld-Tanenbaum Research Institute, Toronto, Ontario, Canada.; 16 Department of Epidemiology, University of Hawaii, Honolulu, Hawaii.; 17 Lunenfeld Tanenbaum Research Institute, Mount Sinai Hospital, University of Toronto, Toronto, Ontario, Canada.; 18 Division of Medical Oncology and Hematology, Princess Margaret Cancer Centre, University Health Network, Toronto, Ontario, Canada.; 19 Department of Pathology, University of Pittsburgh Medical Center, Pittsburgh, Pennsylvania.; 20 University of Chicago Medical Center, Chicago, Illinois.; 21 Roche Tissue Diagnostics, Tucson, Arizona.; 22 Department of Radiology and Machine Intelligence in Medicine and Imaging Center (MI-2), Mayo Clinic Arizona, Phoenix, Arizona.; 23 Department of Oncology, Mayo Clinic, Rochester, Minnesota.; 24 Department of Pathology and Laboratory Medicine, Mayo Clinic, Arizona.

## Abstract

Deep learning may detect biologically important signals embedded in tumor morphologic features that confer distinct prognoses. Tumor morphologic features were quantified to enhance patient risk stratification within DNA mismatch repair (MMR) groups using deep learning. Using a quantitative segmentation algorithm (QuantCRC) that identifies 15 distinct morphologic features, we analyzed 402 resected stage III colon carcinomas [191 deficient (d)-MMR; 189 proficient (p)-MMR] from participants in a phase III trial of FOLFOX-based adjuvant chemotherapy. Results were validated in an independent cohort (176 d-MMR; 1,094 p-MMR). Association of morphologic features with clinicopathologic variables, MMR, *KRAS*, *BRAF^V600E^*, and time-to-recurrence (TTR) was determined. Multivariable Cox proportional hazards models were developed to predict TTR. Tumor morphologic features differed significantly by MMR status. Cancers with p-MMR had more immature desmoplastic stroma. Tumors with d-MMR had increased inflammatory stroma, epithelial tumor-infiltrating lymphocytes (TIL), high-grade histology, mucin, and signet ring cells. Stromal subtype did not differ by *BRAF^V600E^* or *KRAS* status. In p-MMR tumors, multivariable analysis identified tumor-stroma ratio (TSR) as the strongest feature associated with TTR [HR*_adj_* 2.02; 95% confidence interval (CI), 1.14–3.57; *P* = 0.018; 3-year recurrence: 40.2% vs. 20.4%; Q1 vs. Q2–4]. Among d-MMR tumors, extent of inflammatory stroma (continuous HR*_adj_* 0.98; 95% CI, 0.96–0.99; *P* = 0.028; 3-year recurrence: 13.3% vs. 33.4%, Q4 vs. Q1) and N stage were the most robust prognostically. Association of TSR with TTR was independently validated. In conclusion, QuantCRC can quantify morphologic differences within MMR groups in routine tumor sections to determine their relative contributions to patient prognosis, and may elucidate relevant pathophysiologic mechanisms driving prognosis.

**Significance:**

A deep learning algorithm can quantify tumor morphologic features that may reflect underlying mechanisms driving prognosis within MMR groups. TSR was the most robust morphologic feature associated with TTR in p-MMR colon cancers. Extent of inflammatory stroma and N stage were the strongest prognostic features in d-MMR tumors. TIL density was not independently prognostic in either MMR group.

## Introduction

Deep learning (DL) can detect and quantify distinct morphologic features of colon cancers that pathologists may be unable to recognize, require disease-specific expertise, and are time intensive. Among patients with stage III colon cancer, decision-making for adjuvant chemotherapy and its duration (3 vs. 6 months) is determined entirely by T and N stage. Although validated prognostic biomarkers could facilitate adjuvant treatment decisions, very few have been sufficiently validated for clinical application. Importantly, DL may identify biologically important signals embedded in morphologic differences that may underlie tumor subtypes with distinct prognoses (1–3). Other advantages of DL include enhancing pathology interpretive accuracy, increasing efficiency, and reducing interobserver variability (4). To date, however, most DL algorithms have been trained on cancer case series lacking data for DNA mismatch repair (MMR) status which influences pathomorphology and prognosis.

We utilized a segmentation algorithm (QuantCRC) to quantify 15 distinct morphologic features that were previously validated against interpretations by expert gastrointestinal (GI) pathologists (5). Features include stroma and stromal subtypes, tumor grade, signet ring cells, tumor-infiltrating lymphocytes (TIL), tumor budding (TB)/poorly differentiated clusters (PDC), necrosis, and mucin. Precise quantification of these features from a whole slide image would not be possible for pathologists to perform manually. MMR status defines distinct tumor phenotypes with differences in molecular features and patient prognosis. Therefore, we examined the prognostic value of these features in patients with stage III colon cancer from a phase III clinical trial selected by MMR status with the purpose of developing a recurrence prediction model within each MMR subgroup. Results were then validated in an independent cohort.

## Materials and Methods

### Patient Characteristics

The initial cohort consisted of 402 surgically resected stage III colon carcinomas from participants in a phase III adjuvant trial of FOLFOX ± cetuximab where cetuximab did not alter efficacy (NCCTG N0147; Alliance for Clinical Trials in Oncology; ref. 6). All available tumors with deficient (d)-MMR and a similar number of randomly selected proficient (p)-MMR tumors were analyzed (Supplementary Table S1). The validation cohort consisted of 1,275 stage III colon cancers treated with fluoropyrimidine-based adjuvant chemotherapy from participants in the Colon Cancer Family Registry and three separate academic sites (Supplementary Table S2; ref. 7). Each study participant signed a written informed consent for biospecimen use for research. The study was approved by the Mayo Clinic Institutional Review Board and was conducted in accordance Declaration of Helsinki.

### DL Algorithm (QuantCRC)

**FIGURE 1:**
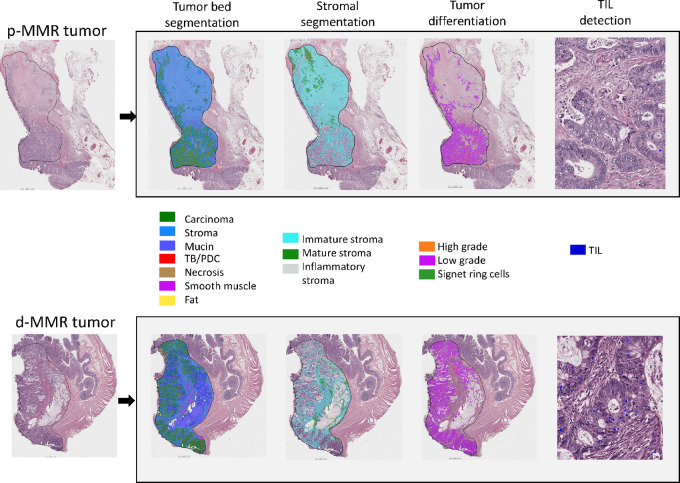
QuantCRC detects tumor morphologic features in four layers. TB/PDC, tumor budding/poorly differentiated cluster; TIL, tumor-infiltrating lymphocytes. https://cloud.aiforia.com/Public/MayoUpmcAiforia_Pai/0z9TK9WQComQSW5MOEo_1KieA8U9KX9oCFbc2SQ-ioM0. To see the QuantCRC analysis click on the image, then click on “analyze,” and then click on the thumbnail of the image.

Tumor blocks were reviewed by a GI pathologist and a representative hematoxylin and eosin (H&E)-stained slide from primary tumors was digitized (VENTANA DP 200 slide scanner; 40X). Whole slide images were submitted to the Aiforia Create DL cloud-based proprietary platform (Aiforia Technologies; https://cloud.aiforia.com/Public/MayoUpmcAiforia_Pai/0z9TK9WQComQSW5MOEo_1KieA8U9KX9oCFbc2SQ-ioM0). QuantCRC was previously developed to segment whole slide images into regions and objects with known pathologic significance (8). After a GI pathologist (R.K. Pai) evaluated each image and outlined the tumor bed, QuantCRC was applied to quantify 15 morphologic features selected and validated by pathologists based on clinical relevance (Table 1). Improved quantification of these known pathologic features may unlock important quantitative data stored in an H&E slide. QuantCRC was previously trained using 24,157 annotations from 1,054 images from 559 colorectal cancers of all stages (8). Convolutional neural networks were used to segment the image in layers (Fig. 1). Tumor bed is first divided into sections: carcinoma, stroma, TB/PDC, mucin, necrosis, fat, and smooth muscle. Second layer divides stroma into mature (densely collagenous areas with scattered fibroblasts, often with parallel collagen fibers), immature (loose, frequently myxoid stroma with scattered fibroblasts and collagen fibers) and inflammatory (dense clusters of chronic inflammatory cells obscuring stromal cell subtypes). The third layer divides carcinoma into signet ring cell, high-grade, and low-grade. TILs within tumor epithelium were identified at fourth layer.

**TABLE 1:** DL-derived tumor morphologic features by DNA MMR status

Morphologic feature, Median (IQR)	p-MMR (*n* = 189)	d-MMR (*n* = 191)	Total (*N* = 380)	*P*-value^a^
Tumor bed^b^ size	78.9 (49.4–116.2)	92.2 (58.0–131.3)	85.3 (52.6–123.0)	0.017
Tumor-stroma ratio	1.00 (0.65–1.49)	1.04 (0.60–1.77)	1.03 (0.61–1.55)	0.412
% High-grade (of tumor area)^c^	3.84 (1.47–9.19)	10.55 (2.52–34.36)	5.03 (1.72–19.06)	<0.001
TIL (count per tumor area)	85.6 (53.0–122.4)	182.4 (86.0–327.2)	105.8 (63.4–215.6)	<0.001
% Mucin (of tumor area)	0.63 (0.13–5.19)	3.26 (0.49–30.53)	1.24 (0.19–19.20)	<0.001
% Necrosis (of tumor bed)	2.99 (1.63–6.40)	3.98 (1.63–10.40)	3.75 (1.63–8.16)	0.082
% Signet ring cell carcinoma (of tumor area)	0.00 (0.00–0.01)	0.006 (0.00–0.09)	0.002 (0.00–0.03)	<0.001
TB/PDS	1.4 (0.6–2.7)	1.4 (0.6–4.3)	1.4 (0.6–3.2)	0.193
%Stroma (of tumor bed)	46.0 (37.3–54.9)	43.7 (31.3–55.5)	44.8 (34.5–55.4)	0.033
% Immature stroma (of tumor bed)	36.1 (29.0–46.0)	28.4 (18.6–38.8)	32.7 (23.0–42.5)	<0.001
% Mature stroma (of tumor bed)	2.52 (1.24–4.96)	2.41 (1.19–4.12)	2.44 (1.23–4.66)	0.351
% Inflammatory stroma (of tumor bed)	3.9 (1.8–6.5)	7.0 (2.7–14.1)	4.8 (2.0–10.1)	<0.001
% Immature stroma (of total stroma)	83.2 (73.9–89.3)	75.3 (54.9–84.4)	78.6 (66.3–86.7)	<0.001
% Inflammatory stroma (of total stroma)	8.2 (3.9–15.3)	15.2 (7.4–36.7)	11.0 (4.8–24.3)	<0.001
% Mature stroma (of total stroma)	5.6 (3.1–11.2)	5.5 (3.0–10.1)	5.6 (3.0–10.4)	0.980

Abbreviations: TB/PDC, tumor budding/poorly differentiated cluster; TIL, tumor-infiltrating lymphocytes.

^a^Kruskal–Wallis *P*-value.

^b^Tumor bed: all the tissue except fat and muscle.

^c^Tumor area: epithelium, tumor budding, and mucin.

TB was defined as a single tumor cell or a cluster of <5 tumor cells at the invasive front; clusters of ≥5 tumor cells without gland formation were defined as PDCs (9).

### Molecular Analysis

Tumors in the initial study cohort were analyzed for MMR proteins by IHC and if indeterminate, microsatellite instability was tested by PCR. In the validation cohort, MMR status was determined by PCR and/or IHC. Tumors were sequenced to identify a mutation in *KRAS* (codon 12 or 13) or *BRAF* c.1799T>A (p.V600E), as described previously (10).

### Statistical Analysis

N0147 cohort was used to build recurrence risk prediction models. Univariable Cox regression was used to assess the relationship between morphologic characteristics and time-to-recurrence (TTR; interval between randomization and first disease recurrence). TTR was chosen as the primary outcome variable because recurrence indicates disease progression, is not impacted by treatment after recurrence, and cancer-specific data for death were not available. Tumor morphologic features were analyzed in relationship to TTR using a data-driven approach. For data analyzed as a continuous variable, Kaplan–Meier curves were constructed with data shown by quartile. Data were analyzed as a dichotomous variable when effects were nonlinear, and quartiles were used for the cut-off points. Only features with a univariate *P* value of <0.05 for association with TTR were included in multivariable models. For morphologic features with a correlation coefficient >0.8 (Spearman rank) with one another, the variable with best *P* value was included in model selection. Backward selection was used to separately build models in p-MMR and d-MMR tumor groups. Model covariates were age, sex, performance status, T/N stage, histologic grade, treatment arm, and *KRAS*, *BRAF*. Two-sided *P* values ≤0.05 were regarded as statistically significant (SAS version 9.4) and were not adjusted for multiple comparisons. The performance of the recurrence risk prediction models was further evaluated witihin each MMR group in the validation cohort using the Harrell concordance index (c-index) and AUC, per methods outlined by Royston and Altman (11). This validation is based on predictions from the trained models with estimates that serve to confirm that the variables were clinically relevant. Data collection and analyses were performed by the Alliance Statistics and Data Management Center.

### Data Availability

The data generated in this study from the N0147 clinical trial can be available by request from the NCTN Navigator mechanism.

## Results

### Patient Characteristics

In the initial study cohort, 380 of 402 tumors met quality control criteria (d-MMR, *n* = 191; p-MMR, *n* = 189). Of these, 183 (48.2%) were female, mean age was 59.4 (SD 12.4) years, and there were 99 recurrences at a median follow-up of 5 years. *BRAF^V600E^* was detected in 111 (30.7%) and *KRAS* mutation in 92 (24.5%) tumors. Patient characteristics stratified by MMR status are shown in Supplementary Table S1. The validation cohort includes 176 tumors with d-MMR and 1,099 with p-MMR.

### Morphologic Features in Relationship to Molecular Variables

Cancers with p-MMR had a higher percentage of immature stroma consistent with desmoplastic reaction in colorectal cancer (Table 1; ref. 12). Among d-MMR tumors, more abundant inflammatory stroma was found which was inversely associated with immature desmoplastic stroma (*r* = −0.87). Tumors with d-MMR versus p-MMR had higher epithelial TIL densities and increased high-grade histology, mucin, and signet ring cells. Tumors with d-MMR and *BRAF^V600E^* had higher epithelial TIL density (*P*-value = 0.01). Among d-MMR tumors, *KRAS* mutation was associated with increased tumor-stroma ratio (TSR; *P*-value = 0.040), lower grade (*P*-value <0.001), and reduced median epithelial TIL density (*P*-value = 0.003). Tumors with p-MMR and *BRAF^V600E^* (vs. nonmutated) had increased mature stroma and less necrosis (both *P*-value ≤0.006).

### Tumor Morphologic Features and TTR by MMR Status

**FIGURE 2:**
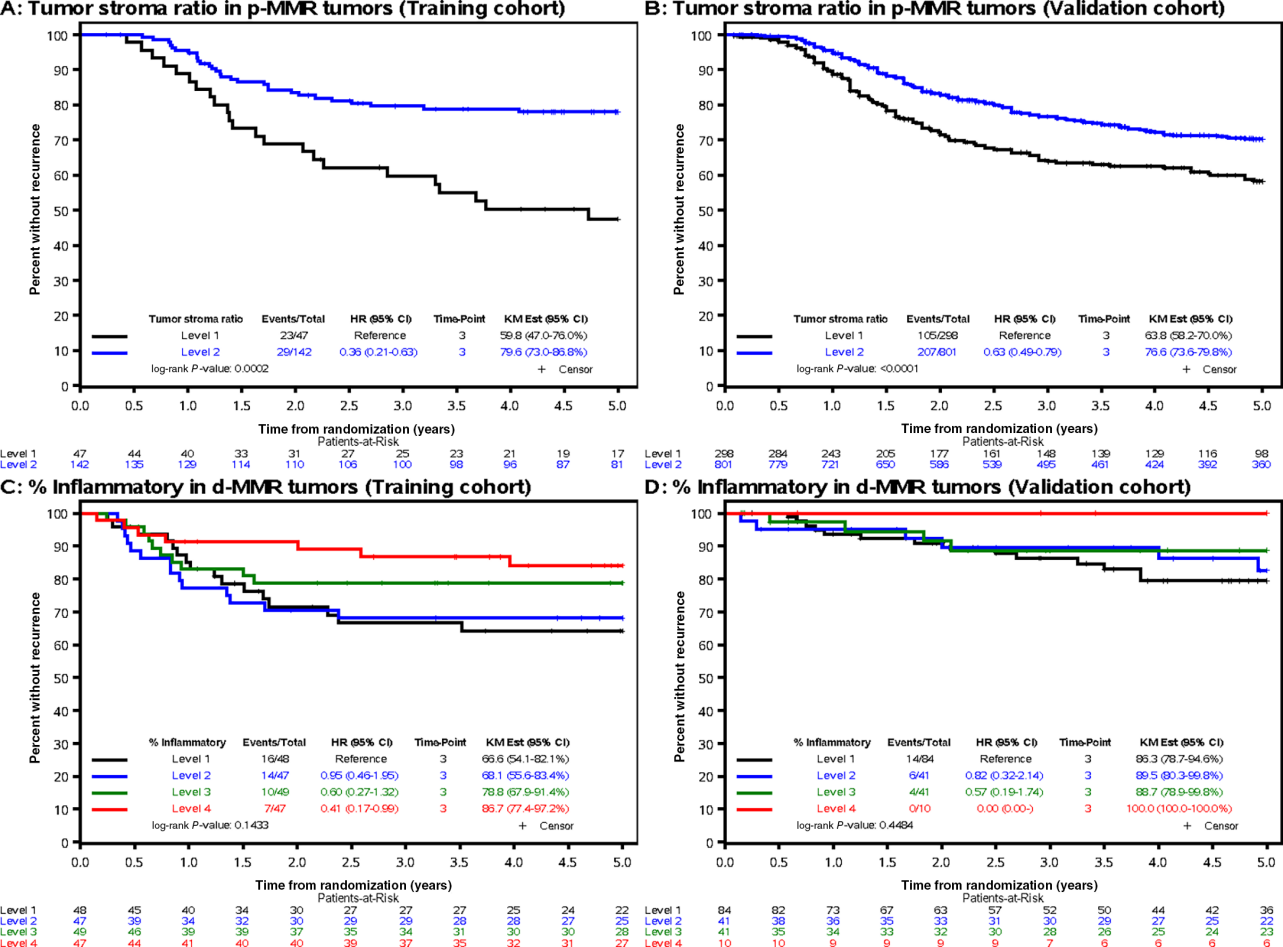
Artificial intelligence–derived morphologic features in stage III colon cancers (NCCTG N0147 trial) that were found to be most strongly associated with patient TTR. Data are shown in Kaplan–Meier plots for TSR among p-MMR tumors in initial cohort (**A**), validation cohort (**B**), and % inflammatory stroma among d-MMR tumors in initial cohort (**C**) and validation cohort (**D**). TSR is shown by level (level 1 < 0.65; level 2 ≥ 0.65). Inflammatory stroma level includes level 1 (<7.38), level 2 (≥7.38 and <15.22), level 3 (≥15.22 and <36.75), level 4 (≥36.75).

Univariate results for the association of morphologic features with TTR by MMR status is shown in Supplementary Tables S3 and S4. In a multivariable analysis among patients with p-MMR tumors, TSR, N stage, T stage, and age were each significantly associated with TTR (Table 2). Of these, TSR was the morphologic feature with the strongest association with prognosis wherein a lower TSR was independently associated with shorter TTR [HR*_adj_* 2.02; 95% confidence interval (CI), 1.14–3.57, *P* = 0.018, Q1 vs. Q2–4] and higher 3-year recurrence rate of 40.2% (Q1) vs. 20.4% (Q2–4; Fig. 2). Among d-MMR tumors, greater extent of inflammatory stroma was independently associated with significantly longer TTR (HR 0.98; 95% CI, 0.96–0.99; continuous; *P* = 0.028, Fig. 2]; 3-year recurrence of 13.3% vs. 33.4% (Q4 vs. Q1). N stage was also significantly associated with TTR (Table 2).

**TABLE 2:** Multivariable models for the prediction of TTR by morphologic features and clinicopathological variables in initial cohort [p-MMR (*n* = 189) or d-MMR (*n* = 191) stage III colon cancers]

Variable	Categorization	HR (95% CI)	*P*-value^a^
p-MMR tumors (52 events/189 cases)			
Nodes	N1 N2	Ref 3.14 (1.71–5.77)	0.001
T Stage	T1 or T2 T3 T4	^b^ Ref 2.56 (1.27–5.26)	0.003
Age	Continuous (5 years)	1.13 (1.003–1.27)	0.042
Tumor-stroma ratio	Q1 Q2–4	2.02 (1.14–3.57) Ref	0.018
d-MMR tumors (47 events/191 cases)			
Nodes	N1 N2	Ref 3.81 (2.06–7.06)	<0.001
Inflammatory stroma	Continuous	0.98 (0.96–0.99)	0.028

^a^Global likelihood ratio *P*-value.

^b^Not estimable due to no events in the “T1 or T2” level.

### Validation Cohort

Among p-MMR tumors, the predictive model showed the ability to discriminate by recurrence status (Harrell c-index 0.62; 95% CI, 0.58–0.65) along with 3-year AUC of 0.76. Among patients with d-MMR tumors, the predictive model had lower discrimination ability (c-index 0.57, 95% CI, 0.46–0.68) along with 3-year AUC of 0.67. Among d-MMR tumors, however, the number of recurrences in relation to sample size was lower in the validation compared with the initial cohort.

Among p-MMR tumors, TSR was confirmed to be the morphologic feature with the strongest association with TTR. Specifically, a lower dichotomous TSR was associated with significantly shorter TTR (HR 1.44; 95% CI, 1.13–1.83; *P* = 0.004; Fig. 2), as were T and N stage (Table 3). The 3-year recurrence rate was 36.2% versus 23.4% (Q1 vs. Q2–4) for low versus high TSR, respectively. Among patients with d-MMR tumors, higher inflammatory stroma was associated with longer TTR [(HR 0.96, 95% CI (0.92–1.01); continuous *P* = 0.087 (Fig. 2; Table 3); 3-year recurrence rate of 0% vs. 13.7% (Q4 vs. Q1)] which showed a similar trend for TTR as in the training cohort.

**TABLE 3:** Multivariable models for the prediction of TTR by morphologic features and clinicopathological variables in the validation cohort [p-MMR (*n* = 1,094) or d-MMR (*n* = 176) stage III colon cancers]

Variable	Categorization	HR (95% CI)	*P*-value^a^
p-MMR tumors (310 events/1,094 cases)			
Nodes	N1 N2	Ref 1.99 (1.59–2.50)	<0.001
T Stage	T1 or T2 T3 T4	Ref 1.39 (0.96–2.01) 2.01 (1.32–3.06)	0.003
Age	Continuous (5 years)	0.98 (0.93–1.02)	0.307
Tumor-stroma ratio	<0.65 ≥0.65	1.44 (1.13–1.83) Ref	0.004
d-MMR tumors (24 events/176 cases)			
Nodes	N1 N2	Ref 1.38 (0.60–3.17)	0.446
Inflammatory stroma	Continuous	0.96 (0.92–1.01)	0.087

^a^Global likelihood ratio *P*-value.

## Discussion

The objective of this study was to examine prognostic value of morphologic features in patients with stage III colon cancer from a phase III clinical trial selected by MMR status with the purpose of developing a recurrence prediction model within each MMR subgroup. We applied a DL algorithm to routine H&E-stained sections of stage III colon carcinomas from a phase III trial of standard FOLFOX-based adjuvant chemotherapy to determine their relative contribution to outcome, and then validated our findings in an external cohort. QuantCRC quantifies 15 distinct tumor morphologic features that had been previously validated against pathologist annotated features in human colorectal cancers (5). Improved quantification of these known pathologic features may unlock important quantitative data stored in an H&E slide. QuantCRC identified a significant increase in immature desmoplastic stroma in p-MMR compared with d-MMR tumors. In contrast, d-MMR tumors had significantly more inflammatory stroma containing chronic inflammatory cells in addition to higher epithelial TILs and established features of high-grade histology, mucin (13), and signet ring cells (14). Interestingly, d-MMR tumors with mutant *KRAS* (13.8%) had a significantly higher TSR and lower epithelial TILs.

Among p-MMR tumors in the initial cohort, lower (vs. higher) TSR was found to significantly and independently predict shorter TTR and higher 3-year recurrence rates of 40.2% versus 20.4%. TSR was validated in an external cohort. In another study where QuantCRC was applied to stage II colon carcinomas, TSR was found to be significantly associated with patient relapse-free survival (5). In human colon cancers, lower TSR (high stroma) was reported to be significantly associated with transcriptomically-determined consensus molecular subtype 4 (CMS4) which exhibits an epithelial-to-mesenchymal transition phenotype (15). The prognostic value of CMS4 was shown to be largely explained by cancer-associated fibroblast (CAF) infiltration score in a multivariable analysis of stage II–III colon cancers (16). As a key component of tumor stroma, CAF functions include collagen deposition and promotion of an immune suppressive tumor microenvironment (TME) which are determinants of progression and metastasis of colorectal cancer (17). Another TME component are TILs which reflect the host antitumor immune response (18, 19) and in several reports, TIL density and Immunoscore have been independent prognostic variables in patients with nonmetastatic colorectal cancer (20, 21). In our cohort, however, TIL density did not remain in the final multivariable model in either p-MMR nor d-MMR tumors.

Multivariable modeling in d-MMR tumors revealed that the extent of inflammatory stroma, encompassing peritumoral lymphocytic reaction and Crohn's like lymphoid reaction (22, 23), was the only morphologic feature that was significantly associated with TTR in the initial cohort. Furthermore, the dichotomized high versus low inflammatory stroma was associated with significantly reduced tumor recurrence at 3 years (13.3% vs. 33.4%). Patients with d-MMR tumors and higher inflammatory stroma showed a trend toward longer TTR in the validation cohort that did not achieve statistical significance, likely due to fewer outcome events compared with the initial cohort (24 vs. 47).

DL may identify biologically important signals embedded in tumor morphologic features which drive distinct prognoses (1–3). In this regard, QuantCRC can quantify distinct tumor regions and has the potential to be used to guide interrogation using spatial multiomics approaches. Other studies in colorectal cancer using DL and an unsupervised approach (1, 3) were shown to provide prognostic information, yet lacked biological interpretability. Strengths of our study include same stage tumors from a clinical trial cohort with uniform treatment, and a multicenter external cohort used to validate our study results. A limitation is that d-MMR tumors in the validation cohort had fewer outcome events than in the initial cohort which limited statistical power for confirmation of the model.

In conclusion, QuantCRC enables quantitation of distinct tumor morphologic features in whole tumor sections that would not be possible for pathologists to quantify manually (5). Moreover, we demonstrate that this DL algorithm applied to routine colon cancer sections can identify those morphologic features that drive patient prognosis within MMR groups. Furthermore, these features may potentially elucidate pathophysiologic mechanisms driving distinct prognoses within MMR groups.

## Supplementary Material

Supplementary Table S1 Supplementary Table S1

Supplementary Table S2 Supplementary Table S2

Supplementary Table S3 Supplementary Table S3

Supplementary Table S4 Supplementary Table S4

## References

[1] Skrede O-J , De Raedt S, Kleppe A, Hveem TS, Liestøl K, Maddison J, . Deep learning for prediction of colorectal cancer outcome: a discovery and validation study. Lancet 2020;395:350–60. 32007170 10.1016/S0140-6736(19)32998-8

[2] Bychkov D , Linder N, Turkki R, Nordling S, Kovanen PE, Verrill C, . Deep learning based tissue analysis predicts outcome in colorectal cancer. Sci Rep 2018;8:3395. 29467373 10.1038/s41598-018-21758-3 PMC5821847

[3] Wulczyn E , Steiner DF, Moran M, Plass M, Reihs R, Tan F, . Interpretable survival prediction for colorectal cancer using deep learning. NPJ Digit Med 2021;4:71. 33875798 10.1038/s41746-021-00427-2 PMC8055695

[4] Tizhoosh HR , Diamandis P, Campbell CJV, Safarpoor A, Kalra S, Maleki D, . Searching images for consensus: can AI remove observer variability in pathology? Am J Pathol 2021;191:1702–8. 33636179 10.1016/j.ajpath.2021.01.015

[5] Pai RK , Banerjee I, Shivji S, Jain S, Hartman D, Buchanan DD, . Quantitative pathologic analysis of digitized images of colorectal carcinoma improves prediction of recurrence-free survival. Gastroenterology 2022;163:1531–46. 35985511 10.1053/j.gastro.2022.08.025 PMC9716432

[6] Alberts SR , Sargent DJ, Nair S, Mahoney MR, Mooney M, Thibodeau SN, . Effect of oxaliplatin, fluorouracil, and leucovorin with or without cetuximab on survival among patients with resected stage III colon cancer: a randomized trial. JAMA 2012;307:1383–93. 22474202 10.1001/jama.2012.385 PMC3442260

[7] Jenkins MA , Win AK, Templeton AS, Angelakos MS, Buchanan DD, Cotterchio M, . Cohort profile: the colon cancer family registry cohort (CCFRC). Int J Epidemiol 2018;47:387–388i. 29490034 10.1093/ije/dyy006 PMC5913593

[8] Pai RK , Hartman D, Schaeffer DF, Rosty C, Shivji S, Kirsch R, . Development and initial validation of a deep learning algorithm to quantify histological features in colorectal carcinoma including tumour budding/poorly differentiated clusters. Histopathology 2021;79:391–405. 33590485 10.1111/his.14353

[9] Fujiyoshi K , Väyrynen JP, Borowsky J, Papke DJ, Arima K, Haruki K, . Tumour budding, poorly differentiated clusters, and T-cell response in colorectal cancer. EBioMedicine 2020;57:102860. 32652320 10.1016/j.ebiom.2020.102860 PMC7347996

[10] Yoon HH , Tougeron D, Shi Q, Alberts SR, Mahoney MR, Nelson GD, . KRAS codon 12 and 13 mutations in relation to disease-free survival in BRAF-wild-type stage III colon cancers from an adjuvant chemotherapy trial (N0147 alliance). Clin Cancer Res 2014;20:3033–43. 24687927 10.1158/1078-0432.CCR-13-3140 PMC4040326

[11] Royston P , Altman DG. External validation of a Cox prognostic model: principles and methods. BMC Med Res Methodol 2013;13:33. 23496923 10.1186/1471-2288-13-33 PMC3667097

[12] Hacking S , Ebare K, Angert M, Lee L, Vitkovski T, Thomas R, . Immature stroma and prognostic profiling in colorectal carcinoma: development and validation of novel classification systems. Pathol Res Pract 2020;216:152970. 32534718 10.1016/j.prp.2020.152970

[13] Lothe RA , Peltomäki P, Meling GI, Aaltonen LA, Nyström-Lahti M, Pylkkänen L, . Genomic instability in colorectal cancer: relationship to clinicopathological variables and family history. Cancer Res 1993;53:5849–52. 8261392

[14] Hewish M , Lord CJ, Martin SA, Cunningham D, Ashworth A. Mismatch repair deficient colorectal cancer in the era of personalized treatment. Nat Rev Clin Oncol 2010;7:197–208. 20177404 10.1038/nrclinonc.2010.18

[15] van de Weerd S , Smit MA, Roelands J, Mesker WE, Bedognetti D, Kuppen PJK, . Correlation of immunological and histopathological features with gene expression-based classifiers in colon cancer patients. Int J Mol Sci 2022;23:12707. 36293565 10.3390/ijms232012707 PMC9604175

[16] Dienstmann R , Villacampa G, Sveen A, Mason MJ, Niedzwiecki D, Nesbakken A, . Relative contribution of clinicopathological variables, genomic markers, transcriptomic subtyping and microenvironment features for outcome prediction in stage II/III colorectal cancer. Ann Oncol 2019;30:1622–9. 31504112 10.1093/annonc/mdz287 PMC6857614

[17] Kobayashi H , Gieniec KA, Lannagan TRM, Wang T, Asai N, Mizutani Y, . The origin and contribution of cancer-associated fibroblasts in colorectal carcinogenesis. Gastroenterology 2022;162:890–906. 34883119 10.1053/j.gastro.2021.11.037 PMC8881386

[18] Yoon HH , Shi Q, Heying EN, Muranyi A, Bredno J, Ough F, . Intertumoral heterogeneity of CD3(+) and CD8(+) T-cell densities in the microenvironment of dna mismatch-repair-deficient colon cancers: implications for prognosis. Clin Cancer Res 2019;25:125–33. 30301825 10.1158/1078-0432.CCR-18-1984 PMC6320300

[19] Lee H , Sha D, Foster NR, Shi Q, Alberts SR, Smyrk TC, . Analysis of tumor microenvironmental features to refine prognosis by T, N risk group in patients with stage III colon cancer (NCCTG N0147) (Alliance). Ann Oncol 2020;31:487–94. 32165096 10.1016/j.annonc.2020.01.011 PMC7372727

[20] Galon J , Costes A, Sanchez-Cabo F, Kirilovsky A, Mlecnik B, Lagorce-Pagès C, . Type, density, and location of immune cells within human colorectal tumors predict clinical outcome. Science 2006;313:1960–4. 17008531 10.1126/science.1129139

[21] Pagès F , Mlecnik B, Marliot F, Bindea G, Ou F-S, Bifulco C, . International validation of the consensus Immunoscore for the classification of colon cancer: a prognostic and accuracy study. Lancet 2018;391:2128–39. 29754777 10.1016/S0140-6736(18)30789-X

[22] Greenson JK , Huang S-C, Herron C, Moreno V, Bonner JD, Tomsho LP, . Pathologic predictors of microsatellite instability in colorectal cancer. Am J Surg Pathol 2009;33:126–33. 18830122 10.1097/PAS.0b013e31817ec2b1 PMC3500028

[23] Jenkins MA , Hayashi S, O'shea A-M, Burgart LJ, Smyrk TC, Shimizu D, . Pathology features in Bethesda guidelines predict colorectal cancer microsatellite instability: a population-based study. Gastroenterology 2007;133:48–56. 17631130 10.1053/j.gastro.2007.04.044 PMC2933045

