# Supplementary material for: Use of Deep Learning to Evaluate Tumor Microenvironmental Features for Prediction of Colon Cancer Recurrence
Source: Cancer Res Commun. 2024 May 23;4(5):1344–50. doi: 10.1158/2767-9764.CRC-24-0031 (PMC11114095; doi:10.1158/2767-9764.CRC-24-0031)
Supplement: Supplementary Table S1 [file crc-24-0031-s01.docx]

*Table S1.* Patient demographic data by MMR status in the training cohort

| Variables ^a^ | p-MMR (n=189) | d-MMR (n=191) | Total (N=380) | *P*-value |
| --- | --- | --- | --- | --- |
| Age, years N (%) |  |  |  | 0.127^1^ |
| <50 | 49 (25.9%) | 37 (19.4%) | 86 (22.6%) |  |
| ≥50 | 140 (74.1%) | 154 (80.6%) | 294 (77.4%) |  |
| Gender, N (%) |  |  |  | 0.001^1^ |
| Female | 74 (39.2%) | 109 (57.1%) | 183 (48.2%) |  |
| Male | 115 (60.8%) | 82 (42.9%) | 197 (51.8%) |  |
| Race, N (%) |  |  |  | 0.463^1^ |
| White | 169 (89.4%) | 175 (91.6%) | 344 (90.5%) |  |
| Non-white | 20 (10.6%) | 16 (8.4%) | 36 (9.5%) |  |
| Ethnicity, N (%) |  |  |  | 0.975^1^ |
| Hispanic or Latino | 8 (4.2%) | 9 (4.7%) | 17 (4.5%) |  |
| Not Hispanic or Latino | 157 (83.1%) | 158 (82.7%) | 315 (82.9%) |  |
| Not Reported or Unknown | 24 (12.7%) | 24 (12.6%) | 48 (12.6%) |  |
| T stage, N (%) |  |  |  | 0.317^1^ |
| T_1_ or T_2_ | 24 (12.7%) | 18 (9.4%) | 42 (11.1%) |  |
| T_3_ | 139 (73.5%) | 173 (80.1%) | 292 (76.8%) |  |
| T_4_ | 26 (13.8%) | 20 (10.5%) | 46 (12.1%) |  |
| N stage, N (%) |  |  |  | 0.902^1^ |
| N_1_ (1-3 nodes) | 112 (59.3%) | 112 (58.6%) | 224 (58.9%) |  |
| N_2_ (≥4 nodes) | 77 (40.7%) | 79 (41.4%) | 156 (41.1%) |  |
| Clinical risk group, N (%) |  |  |  | 0.921^1^ |
| Low (T_1-3_, N_1_) | 97 (51.3%) | 99 (51.8%) | 196 (51.6%) |  |
| High (T_4_ and/or N_2_) | 92 (48.7%) | 92 (48.2%) | 184 (48.4%) |  |
| Histologic grade, N (%) |  |  |  | <0.001^1^ |
| Low | 144 (76.2%) | 84 (44.0%) | 228 (60.0%) |  |
| High | 45 (23.8%) | 107 (56.0%) | 152 (40.0%) |  |
| Performance status, N (%) |  |  |  | 0.376^1^ |
| 0 | 145 (76.7%) | 139 (72.8%) | 284 (74.7%) |  |
| 1/2 | 44 (23.3%) | 52 (27.2%) | 96 (25.3%) |  |
| *KRAS*, N (%) |  |  |  | <0.001^1^ |
| Nonmutated | 121 (64.7%) | 162 (86.2%) | 283 (75.5%) |  |
| Mutated | 66 (35.3%) | 26 (13.8%) | 92 (24.5%) |  |
| Missing | 2 | 3 | 5 |  |
| *BRAF^V600E^*, N (%) |  |  |  | <0.001^1^ |
| Absent | 164 (91.6%) | 87 (47.5%) | 251 (69.3%) |  |
| Present | 15 (8.4%) | 96 (52.5%) | 111 (30.7%) |  |
| Missing | 10 | 8 | 18 |  |

d-MMR, deficient mismatch repair; p-MMR, proficient mismatch repair.

^1^ Chi-square test; ^2^Kruskal-Wallis
