# Supplementary material for: Use of Deep Learning to Evaluate Tumor Microenvironmental Features for Prediction of Colon Cancer Recurrence
Source: Cancer Res Commun. 2024 May 23;4(5):1344–50. doi: 10.1158/2767-9764.CRC-24-0031 (PMC11114095; doi:10.1158/2767-9764.CRC-24-0031)
Supplement: Supplementary Table S2 [file crc-24-0031-s02.docx]

*Table S2.* Patient characteristics in the external validation cohort.

| Variables ^a^ | p-MMR (n=1099) | d-MMR  (n=176) |
| --- | --- | --- |
| Age (years), median (IQR) | 54 (46, 64) | 58 (48, 68) |
| Gender, N (%) |  |  |
| Female | 532 (49%) | 98 (56%) |
| Male | 554 (51%) | 78 (44%) |
| T stage, N (%) |  |  |
| T_1_ or T_2_ | 185 (17%) | 17 (9%) |
| T_3_ | 745 (68%) | 126 (72%) |
| T_4_ | 169 (15%) | 33 (19%) |
| N stage, N (%) |  |  |
| N_1_ (1-3 nodes) | 762 (69%) | 124 (70%) |
| N_2_ (≥4 nodes) | 337 (31%) | 52 (30%) |
| Molecular alteration, N (%) |  |  |
| Nonmutated | 387 (52%) | 47 (39%) |
| Mutated *KRAS* | 298 (40%) | 31 (26%) |
| Mutated *BRAF* | 54 (7.3%) | 41 (34%) |
| Missing | 360 | 57 |
| Center |  |  |
| CCFR | 803 (73%) | 135 (77%) |
| Mayo Clinic | 125 (11%) | 20 (11%) |
| UPMC | 100 (9.1%) | 14 (8%) |
| Mount Sinai | 71 (6.5%) | 7 (4%) |

IQR, interquartile range; CCFR, Colon Cancer Family Registry; UPMC,

University of Pittsburgh Medical Center.
