# Supplementary material for: Use of Deep Learning to Evaluate Tumor Microenvironmental Features for Prediction of Colon Cancer Recurrence
Source: Cancer Res Commun. 2024 May 23;4(5):1344–50. doi: 10.1158/2767-9764.CRC-24-0031 (PMC11114095; doi:10.1158/2767-9764.CRC-24-0031)
Supplement: Supplementary Table S3 [file crc-24-0031-s03.docx]

*Table S3.* Univariate Cox model (linear) of AI-derived morphological features for prediction of patient time-to-recurrence (TTR) in p-MMR stage III colon cancers (training cohort).

|  | Univariate Analysis based on continuous variables | | Univariate Analysis for variables converted to discrete variables ** | |
| --- | --- | --- | --- | --- |
| Variable | Hazard Ratio (95% CI) | Wald P | Hazard Ratio  (95% CI) | Log Rank  p-value |
| TILs per mm^2^ | 0.995 (0.990-1.000) | 0.0611 | NA | NA |
| Tumor/Stroma Ratio | 0.531 (0.315-0.895) | 0.0176 | 2.77 (1.58-4.76)  Ref | 0.002 |
| Tumor bed size | 1.001 (0.996-1.007) | 0.6482 | NA | NA |
| % Stroma of Tumor Bed* | 1.031 (1.012-1.051) | 0.0013 | NA | NA |
| % High-grade | 1.017 (0.995-1.039) | 0.1275 | NA | NA |
| % Mucin | 1.012 (1.000-1.024) | 0.0532 | Ref  1.94 (1.10-3.41) | 0.019 |
| % Necrosis | 0.996 (0.956-1.038) | 0.8520 | NA | NA |
| % Signet ring cell carcinoma | 1.032 (0.859-1.240) | 0.7362 | NA | NA |
| %TB/PDC | 1.039 (1.006-1.074) | 0.0216 | Ref  2.58 (0.90-7.43)  3.48 (1.26-9.57)  5.83 (2.20-15.46) | <0.001 |
| % Immature Stroma of tumor bed * | 1.033 (1.014-1.053) | 0.0007 | NA | NA |
| % Mature stroma of tumor bed | 1.081 (1.024-1.141) | 0.0050 | Ref  2.44 (1.40-4.25) | 0.001 |
| % Inflammatory stroma of tumor bed* | 1.081 (1.024-1.041) | 0.0050 | NA | NA |
| % Immature stroma of total stroma | 1.021 (0.995-1.048) | 0.1128 | NA | NA |
| % Mature stroma of total stroma* | 1.033 (1.003-1.064) | 0.0308 | NA | NA |
| % Inflammatory stroma of total stroma | 0.908 (0.835-0.988) | 0.0168 | NA | NA |

TB/PDC, tumor budding/poorly differentiated cluster; TIL, tumor infiltrating lymphocytes.

* Variable was not included in the model due to correlation with other variables

** Hazard Ratio and P-value are shown for discrete variables included in the model.
