# Supplementary material for: Use of Deep Learning to Evaluate Tumor Microenvironmental Features for Prediction of Colon Cancer Recurrence
Source: Cancer Res Commun. 2024 May 23;4(5):1344–50. doi: 10.1158/2767-9764.CRC-24-0031 (PMC11114095; doi:10.1158/2767-9764.CRC-24-0031)
Supplement: Supplementary Table S4 [file crc-24-0031-s04.docx]

*Table S4*. Univariate Cox Model (linear and categorical) of AI-derived morphological features for prediction of patient TTR in d-MMR stage III colon cancers (training cohort).

|  | Univariate Analysis based on continuous variables | | Univariate Analysis for variables converted to discrete variables ** | |
| --- | --- | --- | --- | --- |
| Variable | Hazard Ratio (95% CI) | Wald P | Hazard Ratio  (95% CI) | Log Rank p-value |
| TILs per mm^2^ | 0.999 (0.997-1.001) | 0.1971 | NA | NA |
| Tumor: Stroma Ratio | 0.980 (0.734-1.308) | 0.8916 | NA | NA |
| Tumor bed size | 1.003 (1.000-1.007) | 0.0882 | NA | NA |
| %Stroma of Tumor Bed | 1.004 (0.987-1.022) | 0.6349 | NA | NA |
| % High-grade | 1.004 (0.992-1.016) | 0.4944 | NA | NA |
| % Mucin | 1.009 (1.000-1.019) | 0.0516 | NA | NA |
| % Necrosis | 1.003 (0.976-1.030) | 0.8404 | NA | NA |
| % Signet ring cell carcinoma | 1.015 (0.994-1.036) | 0.1679 | NA | NA |
| %TB/PDC | 1.000 (0.965-1.036) | 0.9912 | NA | NA |
| % Immature Stroma of tumor bed* | 1.020 (1.000-1.040) | 0.0480 | NA | NA |
| % Mature of tumor bed | 1.049 (0.978-1.126) | 0.1803 | NA | NA |
| % Inflammatory of tumor bed* | 0.967 (0.935-0.987) | 0.0211 | NA | NA |
| % Immature stroma of total stroma* | 1.015 (0.999-1.031) | 0.0653 | NA | NA |
| % Inflammatory stroma of total stroma | 0.981 (0.964-0.998) | 0.0275 | NA | NA |
| % Mature stroma of total stroma | 1.017 (0.987-1.049) | 0.2711 | NA | NA |

TB/PDC, tumor budding/poorly differentiated cluster; TIL, tumor infiltrating lymphocytes.

* Variable was not included in the model due to correlation with other variables

** Hazard Ratio and P-value are shown for discrete variables included in the model.
